# Genetic architecture of wood properties based on association analysis and co‐expression networks in white spruce

**DOI:** 10.1111/nph.13762

**Published:** 2015-11-30

**Authors:** Mebarek Lamara, Elie Raherison, Patrick Lenz, Jean Beaulieu, Jean Bousquet, John MacKay

**Affiliations:** ^1^ Forest Research Centre, and Institute for System and Integrative BiologyUniversité LavalQuébecQCG1V 0A6Canada; ^2^ Canadian Wood Fibre CentreCanadian Forest ServiceNatural Resources CanadaQuébecQCG1V 4C7Canada; ^3^ Canada Research Chair in Forest and Environmental GenomicsUniversité LavalQuébecQCG1V 0A6Canada; ^4^ Department of Plant SciencesUniversity of OxfordOxford0X1 3RBUK

**Keywords:** association genetics, co‐expression network, quantitative genetics, white spruce (*Picea glauca*), wood traits

## Abstract

- Association studies are widely utilized to analyze complex traits but their ability to disclose genetic architectures is often limited by statistical constraints, and functional insights are usually minimal in nonmodel organisms like forest trees.
- We developed an approach to integrate association mapping results with co‐expression networks. We tested single nucleotide polymorphisms (SNPs) in 2652 candidate genes for statistical associations with wood density, stiffness, microfibril angle and ring width in a population of 1694 white spruce trees (*Picea glauca*).
- Associations mapping identified 229–292 genes per wood trait using a statistical significance level of *P *<* *0.05 to maximize discovery. Over‐representation of genes associated for nearly all traits was found in a xylem preferential co‐expression group developed in independent experiments. A xylem co‐expression network was reconstructed with 180 wood associated genes and several known MYB and NAC regulators were identified as network hubs. The network revealed a link between the gene *PgNAC8*, wood stiffness and microfibril angle, as well as considerable within‐season variation for both genetic control of wood traits and gene expression. Trait associations were distributed throughout the network suggesting complex interactions and pleiotropic effects.
- Our findings indicate that integration of association mapping and co‐expression networks enhances our understanding of complex wood traits.

## Introduction

The usefulness of genome‐wide association studies (GWAS) for discovering the genetic basis of complex traits has been shown in many different systems, including, for example, the susceptibility to complex diseases in humans (Altshuler *et al*., 2008) and defense metabolism against herbivory in *Arabidopsis thaliana* (Chan *et al*., 2011). The aim of GWAS is to gain insight into the genetic architecture of such traits through the identification of common genetic variants against the background of random variation in a population (Baranzini *et al*., 2009). One of the challenges of GWAS is that the exceedingly large number of tests and the required stringent statistical criteria typically result in very few associations that exceed a genome threshold of significance after correction for multiple testing (Greenawalt *et al*., 2012). Furthermore, most of the single nucleotide polymorphisms (SNP) variants identified in GWAS provide little or no direct causation into the molecular, cellular or physiological processes underlying the phenotype of interest. Recent studies have shown that innovative approaches may be developed to complement and overcome some of the limitations of association studies and thus optimize the discovery of causative genetic variants (Baranzini *et al*., 2009; Chan *et al*., 2011; Greenawalt *et al*., 2012).

GWAS approaches such as those used in humans (Hirschhorn & Daly, 2005) and some plants (Huang *et al*., 2010; Chia *et al*., 2012) have not been directly amenable for most tree species because of the lack of genomic resources and methods to sample a large enough fraction of existing genome‐wide allelic variation. Therefore, candidate gene approaches were developed as an alternative to GWAS, and consist of selecting genes based on prior knowledge and analyzing them to identify genetic variants for traits of interest. The advantage of this approach was suggested to be related to the rapid decay of linkage disequilibrium (LD) in forest trees which was found to be within 800 bp in loblolly pine (González‐Martínez *et al*., 2006), within 750 bp in Scots pine (Garcia‐Gil *et al*., 2003) and as little as 65 bp in white spruce (Pavy *et al*., 2012). The low levels of LD also suggest that validated marker‐trait associations are likely to be located close to the functional polymorphisms (González‐Martínez *et al*., 2011).

In forest trees, the candidate gene association study (AS) approach has identified SNPs and genes linked to wood and growth traits in many tree species such as *Eucalyptus nitens* (Thumma *et al*., 2009), *Populus* spp, (Ingvarsson *et al*., 2008; Wegrzyn *et al*., 2010; Guerra *et al*., 2013), pines (Dillon *et al*., 2010; Cumbie *et al*., 2011; Jaramillo‐Correa *et al*., 2015) and spruces (Beaulieu *et al*., 2011; Prunier *et al*., 2013). However, the variation in quantitative traits explained by individual SNP markers is generally low and rarely exceeds 5% (Dillon *et al*., 2010; Guerra *et al*., 2013), consistent with multigenic control (Evans *et al*., 2014) and the relatively shallow genomic sampling in most studies to date (< 1% and 10% of estimated gene coding loci per genome) (Nystedt *et al*., 2013; Neale *et al*., 2014).

Controlling for «*false positives*» and assuming «*false negatives*» in GWAS has led to the development of approaches integrating multiple types and sources of data. Protein interaction network‐based pathway analysis was proposed as a strategy to further reduce the large lists of these genes and refine the results of GWAS (Baranzini *et al*., 2009). In *A. thaliana*, network co‐expression approaches were used for the identification of novel genes that affect defense metabolism (Chan *et al*., 2011). Even when the phenotypic effect of each SNP is very small, grouping significant genes according to their function shows that many of these genes contribute together to the same physiological process or a regulatory network (Baranzini *et al*., 2009).

A growing number of studies show that combining association studies, molecular function and expression data could ultimately help increase our understanding of the genomic architecture of complex traits and the genetic basis of variations in trait expression (Baranzini *et al*., 2009; Chan *et al*., 2011; Greenawalt *et al*., 2012). Developing insights into the genomic architecture of complex traits in forest trees will therefore require the testing of more SNPs and genes than reported in most studies to date (Thavamanikumar *et al*., 2013). This will enable the development of global and less biased understanding and the evaluation of pathways and gene networks.

**Figure 1:**
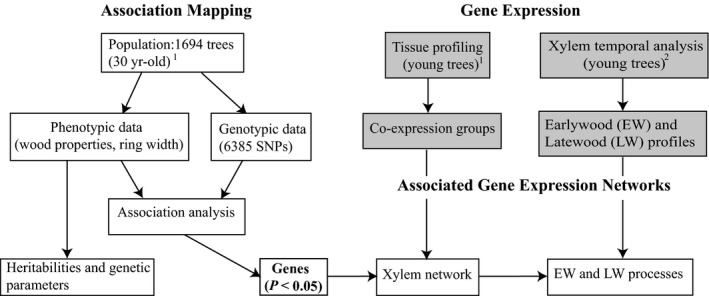
Flowchart of the different analyses and data sources. Shaded boxes represent gene expression data and analyses reported in Raherison *et al*. (2015) which utilized independent trees analyzed under controlled conditions. Focal species: 1, white spruce; 2, white spruce and Norway spruce (*Picea abies* (L.) H. Karst).

Our aim was to delineate the genetic architecture of wood traits in white spruce (*Picea glauca* (Moench) Voss) by testing a large panel of genes with diverse functions and expression profiles (see Pavy *et al*., 2013) and integrate these findings with genome‐wide expression data to shed light onto the underlying gene networks (Fig. 1). The specific objectives of this study were: to identify and compare the genes identified by AS of different wood traits, and determine if they can be attributed to particular biological functions; to study the relationship between the genetic architecture (genes identified by AS) and quantitative genetic parameters; to evaluate whether genes associated with wood traits display preferential expression patterns; and to conduct co‐expression network analysis among genes from the AS as a means of linking genotype‐phenotype associations with cellular processes associated with wood traits.

## Materials and Methods

### Plant material and tissue sampling used for association analysis

The AS population was fully described in Beaulieu *et al*. (2014). Briefly, 1694 trees representing 214 open‐pollinated families (eight trees for most of the families) from 43 provenances were selected in a 30 yr‐old provenance‐progeny test and a 12‐mm increment core was taken at breast height (130 cm aboveground) for wood property determinations. Needle tissue was sampled from the crown for DNA extractions and genotyping.

### Phenotypic data

A wood core from each of the 1694 trees was analyzed at high resolution with the SilviScan technology (Evans, 1999, 2006) at the FPInnovations facilities in Vancouver (Canada). Four wood traits were considered in this study: air‐dry wood density (WD), measured in 25‐μm steps using X‐ray densitometry; microfibril angle (MFA), measured in 1‐mm steps using X‐ray diffractometry; wood modulus of elasticity (MOE) calculated from the densitometry and diffractometry data following Evans (2006); and ring width (RW) determined through information of densitometry profiles. Traits were determined on a ring‐by‐ring basis and data separated into earlywood (EW) and latewood (LW) on the basis of changes in wood density (Beaulieu *et al*., 2011). Averages were calculated for each trait and each tree by weighting individual ring data by its area in an ideal circular disk.

### SNP genotyping

Discovery of the SNPs, genotyping methods and quality control criteria were described previously (Pavy *et al*., 2013; Beaulieu *et al*., 2014). Briefly, for each of the 1694 trees, DNA was extracted from foliage and individuals were then genotyped with the Illumina Infinium HD iSelect bead chip PgAS1 at a rate of 50 ng μl^−1^ previously described by Pavy *et al*. (2013). The SNPs were from candidate genes belonging to 1868 gene families as described by Pavy *et al*. (2013) The candidate genes were selected based on multiple criteria and represented highly diverse functional categories and expression profiles as relevant for growth, phenology, resistance to biotic and abiotic stress, and wood formation (see Supporting Information Methods S1 for more details). Genotypes were obtained for 7434 SNPs in 2813 genes and after quality screening (GenTrain quality score ≥ 0.25, a fixation coefficient |*F*_e_| < 0.50, a minor allele frequency (MAF) ≥ 0.003, a call rate at each SNP locus of ≥ 50%), 6385 SNPs in 2652 genes were retained for subsequent analyses (Beaulieu *et al*., 2014).

### Estimation of quantitative genetic parameters

Genetic parameters of wood traits were estimated using a mixed model approach. Beside the effect for half‐sib families, statistical models were constrained for block and block‐by‐family interactions within the test site. Variance components and co‐variance were estimated with the MIXED procedure in SAS (Littell *et al*., 2006) using restricted maximum‐likelihood (REML). Individual narrow‐sense heritability (*h*_*i*_^*2*^) ,as well as genotypic (*r*_*A*_) and phenotypic (*r*_*P*_) correlations, were determined by a multivariate approach (Lenz *et al*., 2013). The delta method was used for estimation of associated errors (Lynch & Walsh, 1998). More information and formulas are given in Methods S2.

### Association analysis

Missing SNP information was imputed using the RandomForest package v.4.6‐6 in R (Breiman, 2001; R Development Core Team, 2012). Principal component analysis (PCA) was conducted to assess for the presence of population substructure in the population of 1694 trees (Price *et al*., 2006) and a pairwise kinship matrix was calculated to estimate familial relatedness between trees, using all the 6385 SNPs. The association analysis between SNPs and traits were performed with TASSEL (Bradbury *et al*., 2007) v.4.0 standalone. To remove spurious association, the kinship matrix and population structure were used as covariates in the Mixed Linear Model (MLM) (Yu *et al*., 2006). The false discovery rate (FDR) method (Storey & Tibshirani, 2003) was used to correct for multiple testing, whenever it was necessary to compare with results obtained without FDR.

In this study, we focused our expanded analysis at the functional level by considering genes harboring nominally significant SNP associations with wood traits at *P *<* *0.05. By omitting the correction for multiple testing, we aimed to maximize discovery and to gain insight into the genomic architecture and biological processes underlying quantitative traits, rather than identifying a reduced number of statistically stringent associations for phenotype correlation.

### Microarray experiments and gene expression network

Co‐expression group data from two independent gene expression profiling experiments described in Raherison *et al*. (2015) were used to characterize the expression patterns of genes found to be significantly related to wood traits after association testing at *P *<* *0.05 (Fig. 1). For details on the two profiling experiments, datasets and analyses, see Raherison *et al*. (2015). In the present study, two levels of hypergeometric testing were performed to assess the representation in co‐expression groups: (1) over‐ or under‐representation of the entire panel of candidate genes relative to the number of genes in each co‐expression group and (2) separate tests were conducted for over‐ or under‐representation of genes associated with wood traits in gene co‐expression groups (*P *<* *0.05) taking into account the non random distribution of genes tested in the expression groups (for details, see Methods S3).

A gene co‐expression network of 180 significantly associated genes in the two co‐expression groups with xylem preferential expression (M2a and M7b in Raherison *et al*., 2015) was developed based on pairwise gene expression correlations determined between these genes by the Pearson correlation coefficient (*r*) that were calculated using R software (R Development Core Team, 2012). The network was constructed by connecting genes that had an *r*‐value ≥ 0.90 to reduce false connections of weak correlations between genes. The resulting co‐expression networks were visualized using Cytoscape (Shannon *et al*., 2003).

### Functional annotations and enrichment analyses

A BlastX search for the 2652 candidate genes sequences was performed with the Blast2GO, software designed to annotate sequences based on similarity, with an *e*‐value threshold of *e*−10 against the nonredundant protein sequence database. In order to functionally classify these genes, two approaches were used. First, sequences were annotated by assignment of gene ontology (GO) terms and GO terms grouped into GO categories using the plant GO‐Slim terms (Conesa *et al*., 2005). Second, protein families were assigned by using similarities with Pfam families, as described previously (Rigault *et al*., 2011; *e*‐value < *e*−10).

In order to determine whether the different gene lists were enriched for specific GO terms or Pfam domains compared with the overall set of candidate genes tested for associations, enrichment analyses were carried out. Fisher's exact test as implemented in Blast2GO was used to compute the enrichment *P*‐value for each GO term. Enrichment analyses were also performed on identified Pfam domains using Fatiscan at *P‐*value < 0.05 (Al‐Shahrour *et al*., 2007; Medina *et al*., 2010).

## Results

### Quantitative genetic analysis

Individual trait heritabilities, as well as phenotypic and genetic correlations between traits, were estimated for EW and LW traits separately (Table 1a). The narrow‐sense heritability of traits varied between the EW and LW stages and between the different wood traits. In EW, a relatively high level of heritability was observed for WD (*h*_i_^2^ = 0.65), suggesting that this trait is under strong genetic control. Moderate heritability was observed for MFA (*h*_i_^2^ = 0.36) and MOE (*h*_i_^2^ = 0.29). Ring diameter growth, represented by RW, was less heritable (*h*_i_^2^ = 0.18) than physical wood traits, in accordance with expectations that growth is more highly influenced by the environment (Lenz *et al*., 2010). Highly negative genetic and phenotypic correlations were observed between MFA and MOE (*r*_*A*_ = −0.80; *r*_*P*_ = −0.88), as expected given their inverse relationship (Alteyrac *et al*., 2006; Lenz *et al*., 2010). The genetic correlation between WD and mechanical properties (MFA and MOE) was low to moderate. Similarly, low to moderate negative phenotypic correlations were observed between RW and the other wood traits (WD, MOE and MFA).

**Table 1:** (a) Genetic (above diagonal) and phenotypic (below the diagonal) correlations between traits and individual trait narrow‐sense heritabilities (on the diagonal, in bold) in white spruce; (b) genetic (bold) and phenotypic correlations between and within traits and between earlywood and latewood in white spruce

(a)				
Traits^a^	WD	MFA	MOE	RW
Earlywood				
WD	**0.65 (0.09)** b	−0.03 (0.14)	0.56 (0.10)	−0.56 (0.13)
MFA	−0.16 (0.02)	**0.36 (0.08)**	−0.80 (0.06)	−0.37 (0.24)
MOE	0.54 (0.02)	−0.88 (0.01)	**0.29 (0.08)**	−0.04 (0.24)
RW	−0.51 (0.02)	0.22 (0.02)	−0.46 (0.02)	**0.18 (0.07)**
Latewood				
WD	**0.19 (0.07)**	−0.05 (0.22)	0.54 (0.16)	–c
MFA	−0.24 (0.02)	**0.29 (0.08)**	−0.85 (0.05)	–
MOE	0.42 (0.02)	−0.87 (0.01)	**0.35 (0.08)**	–
RW	−0.22 (0.02)	0.39 (0.02)	−0.22 (0.02)	0

(b)					
	Traits^a^	Latewood			
		WD	MFA	MOE	RW
Earlywood	WD	**0.83 (0.12)** b	**0.06 (0.15)**	**0.37 (0.12)**	**–** c
		0.50 (0.02)	−0.29 (0.02)	0.42 (0.02)	−0.19 (0.02)
	MFA	**−0.14 (0.20)**	**0.98 (0.01)**	**−0.85 (0.05)**	**–**
		−0.10 (0.03)	0.94 (0.00)	−0.82 (0.01)	0.15 (0.02)
	MOE	**0.64 (0.18)**	**−0.76 (0.07)**	**0.97 (0.02)**	**–**
		0.39 (0.02)	−0.84 (0.01)	0.89 (0.01)	−0.17 (0.02)
	RW	**−0.62 (0.17)**	**−0.43 (0.25)**	**0.07 (0.22)**	**–**
		0.01 (0.14)	0.24 (0.02)	−0.22 (0.02)	0.21 (0.02)

a Traits: WD, wood density; MFA, microfibril angle; MOE, modulus of elasticity; RW, ring width.

b Error estimates for heritability; genetic and phenotypic correlation estimates are given in parentheses.

c Dashes indicate genetic correlations that could not be estimated.

Compared to the EW, LW heritability estimates were higher for MOE (*h*_i_^2^ = 0.35), lower for MFA (*h*_*i*_^*2*^ = 0.29), lower for WD (*h*_i_^2^ = 0.19) and negligible for RW. Overall, similar patterns were observed in EW and LW for both genetic and phenotypic correlations, with similar strong but negative correlations between MFA and MOE in LW (*r*_*A*_ **= −0.85; *r*_*P*_ = −0.87), and moderate to lower correlations with and among other traits, except for RW, where correlations could not be estimated or were null in LW because family variance components could not be estimated reliably.

All of the traits were clearly under genetic control, both in EW and LW, although the magnitude of heritability estimates varied from low to high among traits (Table 1a). Genetic and phenotypic correlations within traits between EW and LW were high to very high and similar for all wood traits (Table 1b). It is generally assumed that each of the quantitative traits are under multigenic control and the genetic correlations between the different pairs of traits, both within and between EW and LW, suggest what they are controlled by overlapping sets of genes and have similar or distinct genetic architectures. This idea was investigated using an association study (AS) approach.

### Association study

The population structure was explored by principal component analysis (PCA) (Fig. S1) and was found to be weak and only three of the 43 geographic origins were separated, as previously observed using multidimensional scaling with the same data (Beaulieu *et al*., 2014) (Fig. S1). A total of 1543 significant SNP marker‐trait associations were found for all wood traits, with up to 341 SNPs per trait (*P *<* *0.05) (Tables 2, S1, S2). These significant SNPs were distributed among 1120 different genes, that is 42.2% of the candidate genes tested. When using a more stringent criterion (*P *<* *0.01) or correction for multiple testing with the FDR method (*Q *<* *0.20) the total number of significantly associated SNPs dropped to 401 and 11, respectively (Table 2).

**Table 2:** Number of significantly associated single nucleotide polymorphisms (SNPs) and genes after association testing with earlywood and latewood traits in white spruce, and number of significant SNPs after correction for false discovery rate (FDR) (*Q < *0.20)

Traits^a^	Earlywood					Latewood				
	*P *<* *0.05		*P *<* *0.01		*Q *<* *0.20	*P *<* *0.05		*P *<* *0.01		*Q *<* *0.20
	No. SNPs	No. genes	No. SNPs	No. genes	No. SNPs	No. SNPs	No. genes	No. SNPs	No. genes	No. SNPs
WD	338	282	78	70	0	295	257	63	57	2
MFA	330	292	63	59	0	332	290	74	72	2
MOE	329	290	72	68	0	333	287	61	56	0
RW	341	292	78	74	2	269	229	61	53	7

a Traits: WD, wood density; MFA, microfibril angle; MOE, modulus of elasticity; RW, ring width.

**Figure 2:**
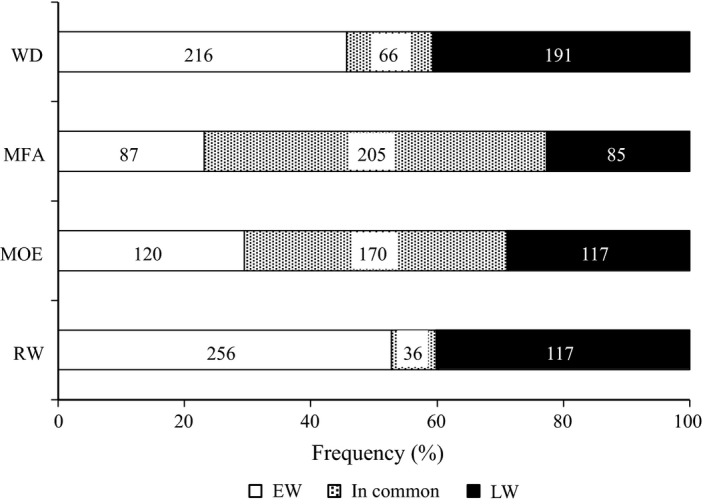
Numbers of significantly associated genes after association tests, specific to earlywood (EW), latewood (LW) or shared (in common) to EW and LW for wood density (WD), microfibril angle (MFA), modulus of elasticity (MOE) and ring width (RW) in white spruce. Numbers in the boxes represent the number of significantly associated genes.

We investigated the 1120 genes with at least one significant association (*P *<* *0.05) to explore their potential roles in the genetic architecture of wood traits. The number of significantly associated genes for each of the wood traits varied from 229 to 292, with many genes associated with more than one trait. Many of the significant genes, that is 430 (38.4%), were found associated with both EW and LW, whereas 362 (32.3%) were associated only with EW and 328 (29.3%) only with LW. Pairwise comparisons of EW and LW sets of significant genes showed that many genes were shared between the two stages of wood formation for MFA and MOE, that is 205 (54%) and 170 (41%) respectively, and fewer genes were shared between EW and LW for WD and RW (Fig. 2). In general, the number of unique genes was higher in EW than in LW.

**Figure 3:**
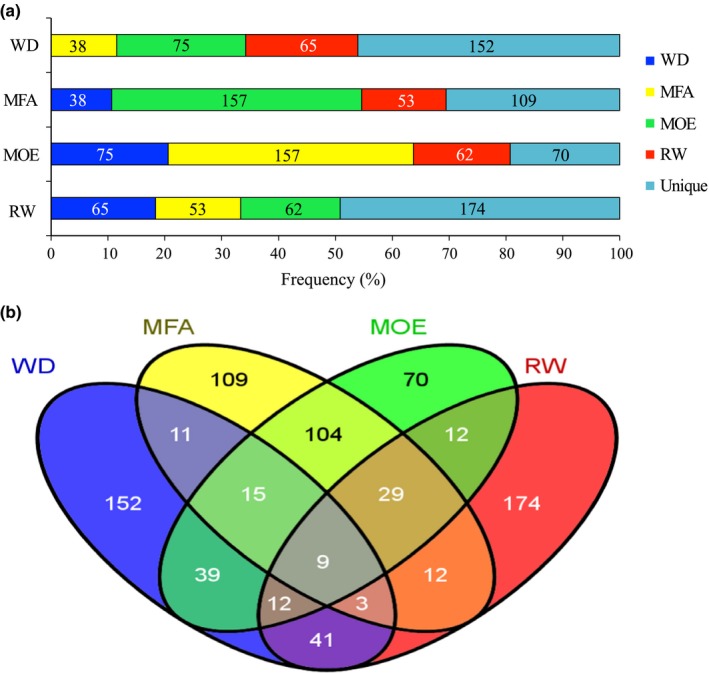
Overlap among sets of significantly associated genes after association testing (*P *<* *0.05) between the different traits as determined for earlywood in white spruce. (a) Pairwise comparisons between traits, showing the numbers shared or unique genes; (b) Venn diagram showing the full extent of overlaps of associated genes between traits. WD, wood density; MFA, microfibril angle; MOE, modulus of elasticity; RW, ring width. For latewood, see Supporting Information Fig. S2.

Comparisons of significant genes for different traits showed a large overlap between MFA and MOE (Figs 3, S2), that is 157 (43%) of significant genes were shared between MFA and MOE in EW. This overlap was consistent with the strong genetic correlation between these same traits (*r*_*A*_ = −0.80; *r*_*P*_ **= −0.88), and it indicates that many of the genes that control MFA also contribute to wood stiffness (MOE). A moderate number of genes overlapped between WD and both MOE and MFA, and fewer with RW. Many more unique genes were observed for WD and RW, suggesting that the set of genes influencing growth is more distinct than those influencing physical wood traits.

Although many genes were associated with at least two traits in EW, only 24 genes were common to the three wood traits (WD, MFA and MOE) and only nine genes were shared between all wood traits and RW (Fig. 3b). Similar results were obtained for LW (Fig. S2). Functional annotations are given in Tables S1 and S2.

### Gene ontology and Pfam enrichment analyses

GO terms and Pfam annotations were analyzed and compared among the subsets of genes significantly associated with the different wood traits. In total, 80% of the 2652 genotyped genes had a predicted function or protein domain and at least one assigned GO term, and the remaining 20% were of unknown function or had no GO term, as reported in Pavy *et al*. (2013). In the set of 1120 genes associated with the different wood properties, 88% had an assigned GO term and 80% had an assigned Pfam.

**Figure 4:**
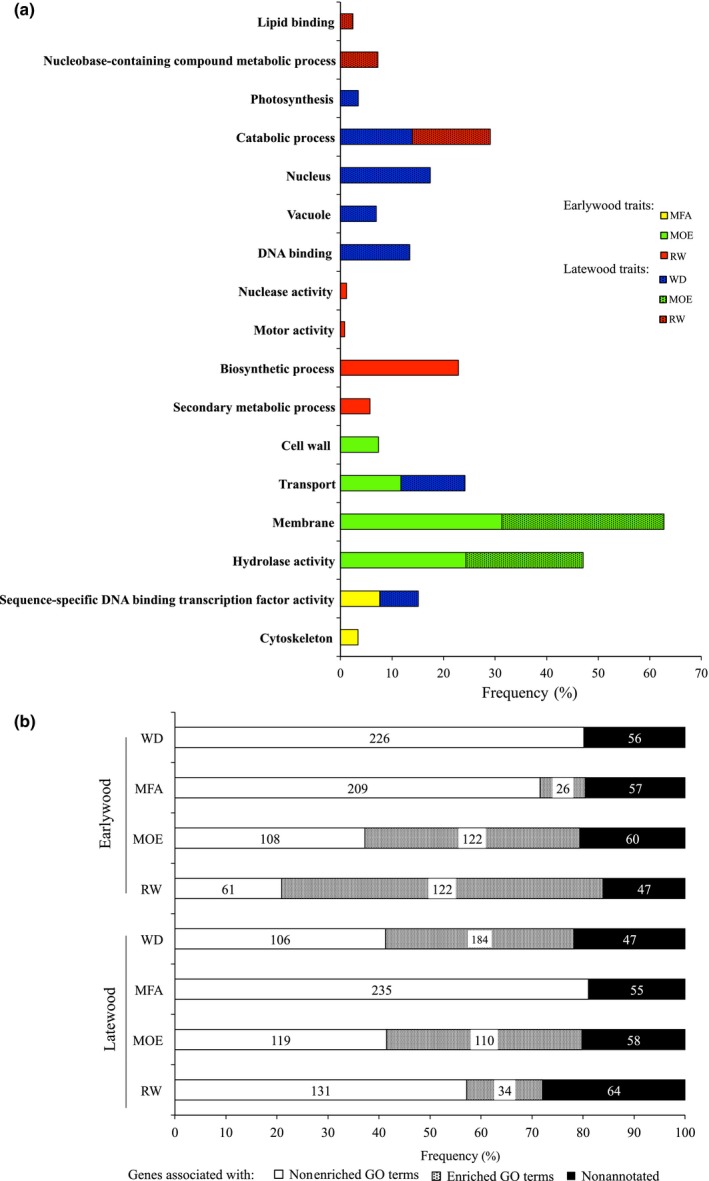
Functional annotations and gene ontology (GO) analysis of significant genes associated with wood traits (WD, wood density; MFA, microfibril angle; MOE, modulus of elasticity; RW, ring width) in white spruce. (a) Enriched GO terms identified for all traits in earlywood and latewood. GO terms include molecular function, biological process and cellular localization categories. Enrichment was determined using Fisher's exact test, *P *<* *0.05. (b) Number of genes (inside bars) and frequencies are according to annotation and GO term classification.

**Figure 5:**
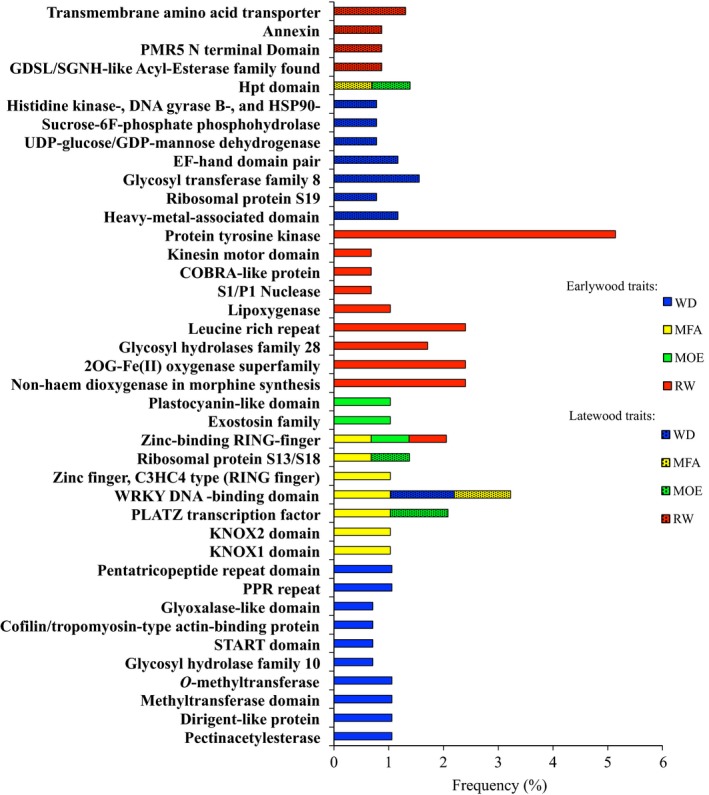
Enrichment analysis of Pfam protein domains in significantly associated genes after association testing in white spruce for all wood traits (WD, wood density; MFA, microfibril angle; MOE, modulus of elasticity; RW, ring width) in earlywood and latewood. Enrichment was determined by Fisher's exact test at *P *<* *0.05.

Enrichment analyses identified 17 GO terms (Fig. 4a) and 40 protein families based on Pfam domains (Fig. 5) that were over‐represented among sets of significant genes for one or several traits (Figs 4, 5). The number of significant genes assigned to enriched GO terms was variable between the traits, and represented up to > 50% of the significant genes (for RW in EW) (Fig. 4a,b). Up to seven enriched GO terms were identified for six of the eight traits considered (Fig. 4a) and up to 10 Pfam domains (Fig. 5) were identified for each of the traits. Overall, both the enriched GO terms and the enriched Pfam domains were very different between traits. Enrichment results were also variable between EW and LW; for example, WD in LW had the most GO terms, whereas WD in EW had none. By contrast, the enrichment analysis for MOE in EW and LW identified similar numbers of significant genes and found two major GO terms in common (transport and hydrolase activity). Together, these data indicated that the genes significantly associated with the different traits are functionally diverse and that the different traits may vary in their levels of functional specialization.

The enriched GO terms and Pfam domains both included functions that may be expected from previous studies of wood growth and development as well as functions not previously reported as linked to these specific traits. Overall, they represented a wide spectrum of cellular processes (e.g. transport, catabolic process, DNA binding; Fig. 4a) and proteins (e.g. transporters, kinases, leucine reach repeat proteins; Fig. 5). They also included processes and enzymes of biochemical pathways related to wood formation, such as cell wall, biosynthetic process, cytoskeleton, secondary metabolic process and several carbohydrate metabolism enzymes. For example, 17 (7.3%) of the genes significantly associated with MOE in EW were assigned to the GO term cell wall and eight (3.4%) of the genes significantly associated with MFA in EW belonged to the GO term cytoskeleton. This overall level of functional diversity is not surprising considering the different cellular events involved in secondary xylem growth and formation (Plomion *et al*., 2001; Carvalho *et al*., 2013).

The Pfam analysis provided a more detailed understanding and enabled the identification of enriched functions for more traits than the GO terms. For MFA in EW, for example, the enriched GO term ‘sequence specific DNA binding transcription factor activity’ was represented by 18 (7.7%) of the significant genes (Fig. 4b), and Pfam domain enrichment analysis specifically identified six classes of transcription factors (Fig. 5). For WD in EW, no enriched GO terms were identified, but nine enriched protein domains were found, including carbohydrate metabolism enzymes (glycosyl hydrolase, pectinacetylesterase), putative secondary metabolism enzymes (methyltransferases, dirigent protein) and a putative regulator of vascular development (START domain) that might be expected for wood traits.

### Gene expression

Co‐expression data were obtained from a separate microarray RNA study on an independent set of young trees which assigned a total of 22 857 genes to 22 co‐expression groups (Raherison *et al*., 2015) and were utilized to further investigate the genes significantly associated with wood traits (Fig. 1, S3). In the present study, we investigated the distribution of candidate genes and of wood associated genes identified by AS among the 22 co‐expression groups by two steps of hypergeometric testing. First, we found that the 2652 candidate genes were significantly over‐represented in five and under‐represented in eight of the co‐expression groups. Second, adjustments were made in the statistical testing to assess over‐ and under‐representations of the significant genes among the co‐expression groups taking into account the nonrandom distribution (Table 3) (for details, see Methods S3). The genes significantly associated with all of the traits except for WD in LW were over‐represented in co‐expression group M2a (Table 3), which is characterized by preferential expression only in secondary xylem and moderate expression in other tissues. The M2a contained 16.0% of the candidate genes tested and up to 26.3% genes associated with wood traits, the highest proportion being for EW RW.

**Table 3:** Distribution in co‐expression groups of white spruce^a^ of total frequencies of genes tested and frequencies of significantly associated genes after association testing

Co‐expression group^b^	Genes tested^c^ (%)	Genes related to earlywood traits^d^ (%)				Genes related to latewood traits^d^ (%)			
		WD	MFA	MOE	RW	WD	MFA	MOE	RW
M1a	7.2***	6.3	7.2	5.3	6.8	8.1	9.0	7.7	6.3
M1b	11.4*	14.2	8.8	11.5	7.2*	12.2	11.3	11.0	12.1
M2a	16.0***	19.6*	22.9**	21.3*	26.3***	15.4	19.5*	20.3*	**20**.0*
M2b	5.0	5.0	3.6	5.3	**8**.0*	4.5	2.7*	4.9	2.4*
M3a	11.6	12.5	6.4**	8.2*	9.6	12.2	6.6**	7.7*	7.7*
M3b	3.0***	4.2	4.4	3.3	2.4	3.6	4.3	3.3	5.3*
M4a	6.6***	3.8*	6.4	6.6	3.6*	5.9	6.3	6.5	5.8
M4b	4.1	4.6	4.0	4.5	4.4	2.7	5.1	3.3	4.4
M5a	**9**.0***	9.2	10.8	8.6	6.4	8.1	9.0	8.5	8.7
M5b	2.6***	2.1	1.2	3.3	2.4	3.6	2.3	2	2.4
M6a	5.3	4.6	5.6	5.7	3.2	5.4	6.6	7.3	5.3
M6b	2***	0.4*	1.6	1.6	1.6	1.8	1.2	1.6	2.3
M7a	3.3	2.1	4	4.5	4	3.2	3.1	3.3	2
M7b	4.2*	5.8	4	2.9	6	4.5	3.9	2.8	3.8
M8a	1	0.4	0.8	0.4	0.8	0.5	0.8	0.8	0.4
M8b	1.3***	0.4	0.8	0.4	0.8	0.5	0.8	0.8	1.0
M9a	1.5	0.8	2.4	1.2	2.4	1.8	2.3	1.6	2
M9b	2.6**	2.5	2.8	4.1	2.0	2.3	3.1	4.5*	3.4
M10a	1.0	0.4	0.4	0	0.8	0.5	0.4	0.4	1.4
M10b	0.8*	1.3	1.6	1.2	0.4	0.5	1.6	1.6	3.0**
M11a	0.2	0.0	0.0	0.0	0.4	1.4	0.0	0.0	0.0
M11b	0.3	0.0	0.0	0.0	0.8	1.4	0.0	0.0	0.0

a Over‐representation of candidate and significant genes in a co‐expression group are represented in bold; under‐represented genes are shown in italic. Significance level: *, *P *<* *0.05; **, *P *<* *0.01; ***, *P *<* *0.001.

b Co‐expression groups were described in Raherison *et al*. (2015) based on tissue profiling across major vegetative tissue types and are shown in Supporting Information Fig. S3. Co‐expression group M2a is characterized by high expression in secondary xylem of shoots and roots and low to moderate expression in other tissues (shoot apex; shoot phelloderm; root phelloderm; young foliage and root tips).

c Over‐ or under‐representation of genes in the given co‐expression group relative to all 2652 candidate genes.

d Over‐ or under‐representation of genes associated with the respective trait (wood density (WD); microfibril angle (MFA); modulus of elasticity (MOE) and ring width (RW)) relative to the total number of candidate genes in the corresponding co‐expression group.

Over‐representation was also found for genes significantly associated with MOE and RW in a few other co‐expression groups with preferential expression in different tissues including secondary xylem and other tissues (MOE and RW in LW) or only others (RW in EW) (Table 3). Genes significantly associated with wood traits were also under‐represented in several co‐expression groups with a variety of profiles, but no clear trend was observed between profiles and traits (Table 3). Under‐representation was found among profiles that have preferential or strong expression in xylem as well as in one or two other tissues (e.g. M3b foliage and xylem; M6b roots and xylem).

### Gene network reconstruction

A co‐expression network analysis was used to identify significantly associated genes with a high level of connectivity, which could indicate a regulatory role. The network was developed with xylem preferential genes (M2a and M7b) significantly associated with wood traits (221 genes in total) (Table 4). Pearson correlations between these genes were obtained from tissue profiling data (Raherison *et al*., 2015) and used at a threshold of *r *≥* *0.9 to construct a network comprised of 180 genes (ranging from 32 to 69 per trait) with correlated expression. Highly connected genes (with the most correlated genes) were designated as hub genes (Table 5). The most highly connected gene in this xylem co‐expression network was *PgNAC‐7*, which was connected to 50 other genes and was recently shown to be strong candidate for the regulation of secondary cell wall formation spruce (Duval *et al*., 2014). The other 20 top‐ranking hub genes included another NAC transcription factor (*PgNAC‐8*), three MYB transcription factors and several biosynthesis enzymes of secondary cell wall polysaccharides (e.g. cellulose) and lignin correlated with *PgNAC‐7* (Table 5).

**Table 4:** Numbers of wood associated genes with xylem preferential expression and subsets selected to reconstruct a co‐expression network

Traits^a^	Xylem preferential genes (M2a and M7b)		Genes used to construct network (*r* ≥ 0.9 with at least one other gene)		Genes connected to MYBs and NACs (*r* ≥ 0.9)	
	EW	LW	EW	LW	EW	LW
WD	61	44	50	32	24	18
MFA	67	60	54	47	32	26
MOE	59	57	49	48	25	25
RW	81	49	69	38	42	23

a Traits: WD, wood density; MFA, microfibril angle; MOE, modulus of elasticity; RW, ring width.

**Table 5:** The 20 hub genes and their functions in the wood co‐expression network in white spruce

GenBank accession number	Cluster ID^a^	Gene name^b^	Degree^c^	Rank^d^	Functional annotation
BT102049	GQ0165_B14	NAC‐7*	50	1	NAC‐domain transcription factor, NAC 007
BT108136	GQ03117_E18	MYB8*	42	2	MYB domain protein
BT117395	GQ03818_K09	*SHM4*	42	2	Serine hydroxymethyltransferase 4
BT109520	GQ03209_H09	*DUF579*	41	3	Protein of unknown function
BT111350	GQ03236_G10	PRR‐2*	41	3	Pinoresinol reductase 2
BT116706	GQ03805_C07	DHS‐2*	40	4	3‐deoxy‐d‐arabino‐heptulosonate 7‐phosphate synthase
BT118944	GQ04012_D01	*Unknown*	39	5	Protein of unknown function
BT102121	GQ0166_N10	LPTHIO‐1*	38	6	Lysophospholipase 2
BT106709	GQ03008_L07	*GATL7*	38	6	Galacturonosyltransferase‐like 7
BT106091	GQ02902_M04	*PK*	37	7	Protein kinase protein with adenine nucleotide alpha hydrolases‐like domain
BT106204	GQ02904_O19	*DUF716*	37	7	Protein of unknown function
BT106749	GQ03009_M04	*RING/U‐box*	37	7	RING/U‐box superfamily protein
BT107152	GQ03103_F19	*TPR_Like*	37	7	Tetratricopeptide repeat domain‐containing protein
BT101192	GQ0072_B14	*PR*	34	8	Pathogenesis‐related thaumatin‐like protein
BT106820	GQ03011_G09	*MAP65‐1*	34	8	Microtubule‐associated proteins 65‐1
BT105950	GQ02830_G18	*IQD2*	33	9	IQ‐domain 2
BT106875	GQ03012_K10	*LRR‐PK*	33	9	Leucine‐rich repeat protein kinase family protein
BT117303	GQ03816_N01	*SEP*	33	9	Subtilisin‐like serine endopeptidase family protein
BT116853	GQ03807_P11	*LRR*	32	10	Leucine‐rich repeat protein kinase family protein
BT107883	GQ03113_N22	MYB4*	31	11	MYB domain protein

a From Rigault *et al*. (2011).

b Gene name in the network.

c Degree, defined as the link numbers one gene has to the other within the network, based on M2a and M7b co‐expression groups.

d Rank, based on their degree of connectivity within the network whereby genes with similar degree of connectivity have the same rank; *Pg‐gene name in white spruce (*Picea glauca*) according to Duval *et al*. (2014).

**Figure 6:**
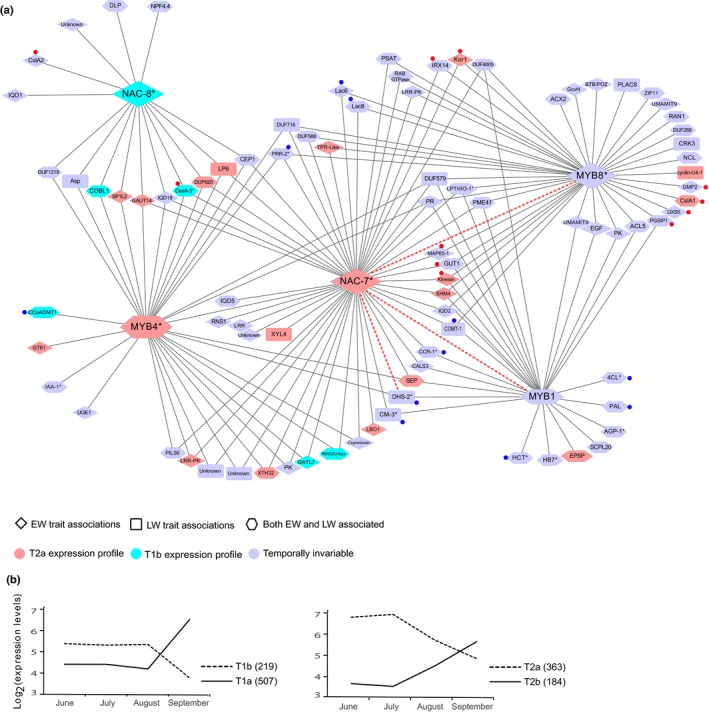
Co‐expression network and expression profile of 93 significantly associated genes highly expressed in the xylem of white spruce. (a) Gene co‐expression network (*r *≥* *0.9) of genes highly expressed in secondary xylem (co‐expression groups M2a and M7b from Raherison *et al*. (2015)), showing genes connected to NAC and MYB transcription factors. *NAC‐7, MYB1, MYB4* and *MYB8* genes were previously linked to secondary cell wall formation in white spruce (Bomal *et al*., 2008, 2014; Duval *et al*., 2014) but not *NAC‐8*. Hub genes listed in Table 5 have a thick border. Lines connecting genes indicate correlated expression; lines of dashed red arrows indicate trans‐activation by *NAC‐7*gene (Duval *et al*., 2014). Functional annotations: involved in lignin biosynthesis (blue dots), cellulose and xylan biosynthesis (red dots). Full gene names and their functions are provided in Table S3. Pg‐gene names marked with an asterisk (*) are according to Duval *et al*. (2014). EW, earlywood; LW, latewood. (b) Expression profiles of temporally variable genes in secondary xylem of white spruce (*Picea glauca*) and Norway spruce (*Picea abies*) (Raherison *et al*., 2015). T2a co‐expression group that varied progressively from June to mid‐July and fall down from August to September, T1a co‐expression group that characterized by their stable transcript levels in June and July and increased dramatically from August to September (Raherison *et al*., 2015).

For clarity, we represented the resulting xylem network by showing the 93 genes that were connected to either one of NAC‐7, NAC‐8, MYB1, MYB4 or MYB8 (Fig. 6a). We examined the trait associations of the genes in the network in more detail by considering whether the associations were found with EW or LW traits and the type of traits that were associated with each gene.

First, a large majority of the genes (86.5%) were associated with one or several EW traits, including 43.8% and 42.7% associated uniquely with EW traits or with both EW and LW traits, respectively. By contrast, only 13.5% of the genes were uniquely associated with LW traits. We tested whether this pattern could be linked to expression by using data from a temporal variation study comparing EW and LW expression from Raherison *et al*. (2015) (Fig. 6b). Nearly a third of the network genes (29.6%) were preferential to EW, none were preferential to LW and 70.4% were nonvariable (Fig. 6a). Three of the transcriptional regulators, that is *PgNAC‐7*,* PgNAC‐8* and *PgMYB4*, were preferentially expressed in EW, whereas *PgMYB1* and *PgMYB8* did not vary temporally.

**Figure 7:**
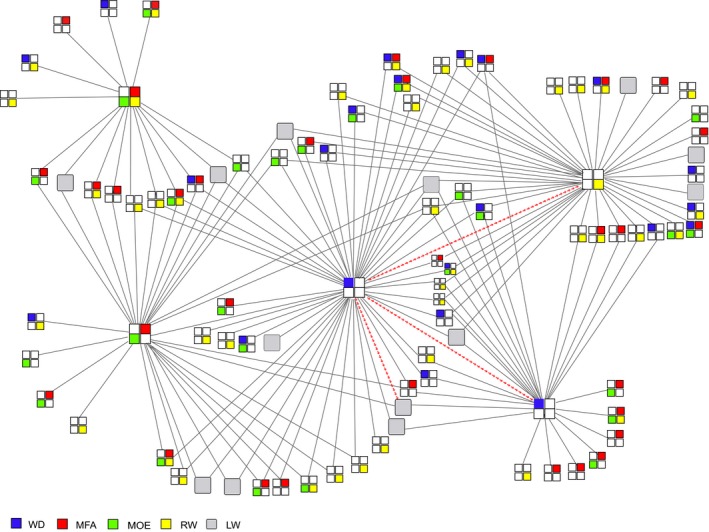
Map of the earlywood (EW) trait associations (wood density (WD); microfibril angle (MFA); modulus of elasticity (MOE) and ring width (RW)) across the gene co‐expression network. All of the 93 genes in the network are shown, 80 genes have EW traits associations (colored boxes) and 13 genes only have latewood (LW) associations (gray boxes).

Second, we mapped the trait associations back onto the network. Using the xylem network (Fig. 6) as a template, the EW trait associations for each gene (Fig. 7) showed that the different traits are distributed throughout the network with no strong pattern or organization. However, NAC7 which is specifically associated with WD is also linked to a majority of WD associated genes including *MYB1*. The NAC8 and *MYB4* genes were associated with both MFA and MOE but other genes associated with those two traits were found throughout the network.

## Discussion

### Genes associated with physical wood traits

Association mapping based on candidate genes has been used to link single nucleotide polymorphisms (SNP) in candidate genes with complex traits and develop an understanding of the molecular genetic basis of growth and wood quality in conifers (González‐Martínez *et al*., 2007, 2011; Beaulieu *et al*., 2011). The SNP‐by‐SNP association approach has shown that the effect of each SNP on the total phenotypic variation is generally low, which is expected for multigenic control and may also suggest complex gene interaction effects (González‐Martínez *et al*., 2008; Prunier *et al*., 2013).

In the present study, we tested for marker‐trait associations based on a total of 6385 polymorphic SNPs in 2652 different candidate genes. Hundreds of SNPs and candidate genes were identified at a nominal threshold of *P *<* *0.05 for each of the traits tested, but very few remained significant after false positive correction with an false discovery rate (FDR) of 0.20. Most studies testing for associations between complex traits and SNPs in forest trees have shown that the vast majority of associations do not hold up to correction for multiple testing (Beaulieu *et al*., 2011; Chhatre *et al*., 2013). This problem may have several different causes and result in the rejection of associations and genes that contribute to the trait variation but with very small or variable effects. As the objective of this study was to uncover the genetic architecture of quantitative variation in wood traits, we aimed to discover genes and expression networks and not predict phenotypic variances. The proportion of significant gene SNPs at *P *<* *0.05 reported here for each trait is similar to that obtained in previous association studies using mixed linear model (MLM) methods, in white spruce (Beaulieu *et al*., 2011) and in loblolly pine (Chhatre *et al*., 2013).

### Relationship between genetic architecture and quantitative genetic parameters

We estimated heritability and genetic parameters to improve our understanding of the genetic architecture underlying complex phenotypes. Intraspecific genetic variation is well known to influence wood features such as density and fiber length in forest trees (Lenz *et al*., 2010; Stackpole *et al*., 2010). Here, we reported heritability and genetic parameter estimates for wood traits from determinations in 1694 trees belonging to 214 open‐pollinated families and obtained very similar results to those of Lenz *et al*. (2010), who studied 25 of the same families replicated in three distinct ecological regions. Our results also agree with numerous other studies in forest trees, indicating that wood properties are generally under moderate to strong additive genetic control in contrast to growth, which is under lower genetic control (Stackpole *et al*., 2010).

It may be expected that genetic and phenotypic correlations between wood traits are underpinned by genes and gene networks that are shared among traits (Mackay *et al*., 2009) but this has not been directly tested for wood traits in forest trees. In the present study, strong genetic and phenotypic correlations were observed between microfibril angle (MFA) and wood modulus of elasticity (MOE), which is consistent with previously described relationships between MFA and MOE in conifers such as *Pinus taeda* (Cramer *et al*., 2005). We also observed negative correlations between diameter growth rate (i.e. RW) and physical wood traits, which is congruent with other reports indicating a negative genetic relationship between growth and wood density (WD) **(**Apiolaza *et al*., 2005; Li & Wu, 2005).

We observed that the number of associated genes which were shared between traits or between growth stages was directly proportional to the magnitude of their genetic and phenotypic correlations. The number of shared genes indicated higher similarity between physical wood traits such as MOE and MFA than with WD and RW (Table 1; Fig. 3a). This observation may indicate that significant SNPs at a level of *P *<* *0.05 are informative of the underlying genetic architecture, even though the majority of SNPs would have been discarded by FDR correction. The genetic correlation results, together with the strong overlap among sets of associated genes further suggest that MFA and MOE have similar genetic architectures in white spruce and that the genes may have pleiotropic effects. We also observed that, when comparing early and late stages for a given trait, the number of overlapping genes ranged from only 8% (for RW) to 55% (MOE, MFA), indicating that the genetic architectures of physical wood traits are less variable over the course of a growth season than the amount of wood formed (Fig. 2).

### Gene functions associated with physical wood traits

A central goal of this study was to assess the biological functions and roles of genes identified by association analysis of wood traits. The large number of genes tested and identified enabled a broad analysis of functional annotations compared to several of the previous studies in forest trees (Beaulieu *et al*., 2011), which represents a significant step toward the goal of understanding the genetic architecture of these complex traits.

The GO term and Pfam enrichment analyses identified enriched biological processes and protein families for the different traits. A range of different categories and protein families are represented among the genes associated with wood traits, which is consistent with the complex nature of wood formation (Carvalho *et al*., 2013). Wood properties are determined by the amount and proportion of secondary cell wall materials deposited during secondary xylem growth and the ultrastructure of cellulose polymers assembled into microfibrils in the thickened cell walls. The major constituents of wood are the complex carbohydrates cellulose and hemicelluloses, and lignin, a phenolic polymer that impregnates the carbohydrate matrix (Plomion *et al*., 2001). Our results are indicative of the wide variety of molecular functions and processes that lead to the formation and the genetic variation of secondary xylem. The lack of overlap in enrichment results between wood traits and seasonal growth phases (early wood (EW) and late wood (LW)) may indicate distinct functional signatures associated with the different traits despite the considerable overlap between some of the gene lists.

The mechanical properties of wood and fibers are determined to a significant extent by MFA, which directly explained up to 70% of the variation in wood stiffness as determined by MOE (Alteyrac *et al*., 2006). In Eucalyptus, MFA alone has been estimated to account for 86% of the variation in wood stiffness (Evans & Ilic, 2001). We report a genetic correlation of −0.80 and a phenotypic correlation of −0.88 between MFA and MOE in white spruce. The negative relationship is expected because a small MFA leads to higher stiffness (MOE) such that the correlations are negative. Several genes and their allelic variants have previously been found to affect MFA and wood stiffness (Li *et al*., 2011); however, the molecular mechanisms controlling microfibril orientation and mechanical stiffness are largely uncharacterized. In the present study, we found that genes involved in cytoskeleton development and several transcription factors were over‐represented among genes significantly associated with EW MFA. The cytoskeleton plays a key role in the establishment of cell wall ultrastructure and resulting mechanical properties of the xylem tissue (Ryden *et al*., 2003; Fletcher & Mullins, 2010). These genes are involved in the two main types of cytoskeletal polymers: actin filaments and microtubules (Fletcher & Mullins, 2010). The complex architecture of the cytoskeleton appears to involve the effects of several transcription factors, according to our Pfam enrichment results (Fig. 5) and other authors (Zhong *et al*., 2011; Mizrachi *et al*., 2012).

### Expression profiles and reconstruction of a wood formation gene network

The 2652 candidate genes in the present AS were very diverse, belonged to 1868 gene families and were selected based on multiple criteria. The candidate genes were categorized according to findings from a recent large‐scale analysis of gene expression profiles in white spruce tissues (Raherison *et al*., 2015). Their distribution across the 22 different co‐expression groups defined by Raherison *et al*. (2015) was different from that expected by a chance alone, that is they were over‐ or under‐represented in 16 of the 22 co‐expression groups. The subset of 1120 genes significantly associated with wood traits showed further over‐ and under‐representations beyond that observed in the entire set of candidate genes but only in a few of the co‐expression groups. They were almost exclusively over‐represented in the co‐expression group (M2a) with secondary xylem preferential expression. This finding led us to construct a gene expression network with wood traits associated genes that were preferential expressed in secondary xylem (M2a and M7a) and had strongly correlated tissue expression profiles. This unbiased approach to select genes identified by AS reduced the total number of genes investigated from 1120 to 180, of which 93 were connected to one of five NAC and MYB transcriptional regulators (Fig. 6a).

The *PgNAC‐7* gene associated with WD in EW was the most highly connected hub gene. This observation is consistent with the report of Duval *et al*. (2014), that *PgNAC‐7* is a master regulator of secondary cell wall biosynthesis in conifer xylem and is functionally similar to the *Arabidopsis* gene *VND6* based on co‐transfection (promoter activation), expression and sequence similarity results. Raherison *et al*. (2015) also reported that *PgNAC‐7* is a major hub gene that is preferentially expressed during the formation of EW.

The MYB genes *MYB1*,* MYB4* and *MYB8* are part of the network and have been shown to be functionally linked to secondary cell wall formation and lignin biosynthesis through over‐expression experiments and electrophoretic mobility shift assay binding to AC elements in gene promoters *Picea glauca* (Bomal *et al*., 2008, 2014) and in *Pinus pinaster* (Craven‐Bartle *et al*., 2013). Similar results were also reported for loblolly pine *MYB1* and *MYB4* (Patzlaff *et al*., 2003a,b). *PgNAC‐7* and these three MYBs were proposed by Duval *et al*. (2014) to form a regulatory network similar to the SND1 network defined in *Arabidopsis* (Zhong *et al*., 2006) that is conserved in poplar (Lin *et al*., 2013; Wang *et al*., 2014). The regulation of lignin biosynthesis by members of the conserved SND1 transcriptional network has been well documented in conifer trees (Patzlaff *et al*., 2003a,b; Bomal *et al*., 2008, 2014). The lignin biosynthetic pathway is well described at the molecular level and at least 10 gene families are involved in the pathway of monolignol biosynthesis in trees (Lu *et al*., 2010). Most of these genes are present in the network described in the present study and were connected to one of the *PgMYBs* and to *PgNAC‐7*. For example, *PgHCT*, PAL and *Pg4CL* were connected to *PgMYB1* in agreement with functional characterizations showing that the *MYB1* gene product positively regulates genes involved in the phenylpropanoid pathway (Bomal *et al*., 2008). The other genes that were connected to *PgMYB8*,* PgMYB4* and *PgNAC‐7*, suggesting that their regulation depends on several transcription factors. These results support observations that several different MYBs may act in concert to regulate different portions of phenylpropanoid metabolism and lignin biosynthesis at different times in conifer trees (Patzlaff *et al*., 2003a; Bomal *et al*., 2014).

The proteins and cellular processes involved in cellulose and xylan biosynthesis are also well described in plants (see Mizrachi *et al*., 2012 for review) but few reports have documented gene networks and the transcriptional regulators underlying their synthesis, especially in trees. Our network results indicate that *PgNAC‐8* is connected to *PgCesA3* and *CslA2*, suggesting that it could be involved in the regulation of cellulose biosynthesis together with *PgNAC‐7*. The *PgNAC‐8* gene was identified in Duval *et al*. (2014) but had not been directly linked to xylem formation. The present study identified *PgNAC‐8* as associated with MFA and MOE and as part of a network of several genes associated with the same and other wood traits. Recent work by Duval *et al*. (2014) identified over 20 different NACs in *P. glauca* which have not been functionally characterized. Their phylogenetic analysis point to five potential candidate orthologs (*PgNAC‐3*,* 8, 17, 19, 25*) for the *AtSND2*,* 3* genes which regulate complex carbohydrates during secondary cell wall formation (Zhong *et al*., 2010). Together the expression profile of *PgNAC‐8* and its association with several EW and LW properties make it a strong candidate ortholog for this function in spruce.

Both *PgNAC‐7* and *PgNAC‐8* were preferentially expressed during the formation of EW, but the *PgNAC‐8* transcript levels were maintained later in the growth season until August, compared to *PgNAC‐7*, whose expression decreased before August (Raherison *et al*., 2015). In *Arabidopsis*, SND2 and SND3, the closest homolog to *PgNAC‐8*, have been shown to regulate secondary cell wall CesA genes (Zhong *et al*., 2008). Taken together, these results point to a network involving NAC and MYB regulators that regulate secondary cell wall properties and influence EW traits most strongly, which was suggested to change over the course of a growing season.

Our analysis yielded fewer insights into the formation and properties of LW. Two genes among the top 20 hub genes (Table 5) were of unknown function and were associated only with LW traits (MOE and RW) and were connected to all of the other top 20 hub genes. They may be involved in an LW gene network, but functional studies and further expression analyses will be necessary to determine their molecular roles.

Mapping the trait associations back onto the xylem expression network provides a clear indication that the genes effects are distributed across the network, suggesting complex genetic and functional relationships that merit further dissection. For example, functional relationships could be assessed using transient assays of transcription factor – promoter interactions as reported recently in spruce (Duval *et al*., 2014). At the genetic level, candidate gene associations could be explored further by combining haplotype analyses and expression levels in the same trees.

## Conclusion

We carried out comparative and combined analyses of genes associated with wood traits using a significance threshold of *P *<* *0.05. The outcomes provided new insights into the underlying multi‐loci genomic architectures. Comparative analyses of the different traits revealed: (1) conservation in the makeup of associated genes that was proportional to genetic and phenotypic correlations between traits and stages; (2) much diversity and low conservation in over‐representation GO terms and protein families; and (3) well conserved expression profiles. Co‐expression analysis identified a wood formation wall network strongly linked to EW traits and an unexpected NAC gene. Our results reveal links between genetic architecture and co‐expression networks underlying wood properties.

## Author contributions

M.L., J.M., J.Be. and J.Bo. planned and designed the research. E.R. performed the expression data analyses. P.L. performed the quantitative analyses of wood traits. M.L. carried out the association mapping, analyses of wood associated genes and network reconstructions. M.L. drafted the manuscript. J.Be., J.Bo. and J.M. revised the manuscript.

## Supporting information

Please note: Wiley Blackwell are not responsible for the content or functionality of any supporting information supplied by the authors. Any queries (other than missing material) should be directed to the *New Phytologist* Central Office.

**Fig. S1 **Plots of the 1694 white spruce trees on the plane of the two‐first eigenvectors derived from the principal component analysis (PCA).
**Fig. S2 **Overlap among sets of significantly associated genes (*P *<* *0.05) between the different traits as determined for latewood.
**Fig. S3** Gene co‐expression groups in white spruce (*Picea glauca*) according to Raherison *et al*. (2015) that were used for network reconstructions.

**Table S1** Genes significantly associated with EW traits and their functions
**Table S2** Genes significantly associated with LW traits and their functions
**Table S3** The 93 selected genes that were connected to NAC‐7, NAC‐8, and to MYB1, MYB4 and MYB8 in the co‐expression network and their functions
**Methods S1 **Candidate genes selection.
**Methods S2 **Information and formulas used for estimation of quantitative genetic parameters.
**Methods S3 **The hypergeometric test used for the evaluation of the over‐ and under‐representation of candidate and significant genes in the co‐expression groups.
