# Supplementary material for: Genetic architecture of wood properties based on association analysis and co‐expression networks in white spruce
Source: New Phytol. 2015 Nov 30;210(1):240–55. doi: 10.1111/nph.13762 (PMC5063130; doi:10.1111/nph.13762)
Supplement: Supplementary file 1 — Fig. S1 Plots of the 1694 white spruce trees on the plane of the two‐first eigenvectors derived from the principal component analysis (PCA). Fig. S2 Overlap among sets of significantly associated genes (P < 0.05) between the different traits as determined for latewood. Fig. S3 Gene co‐expression groups in white spruce (Picea glauca) according to Raherison et al. (2015) that were used for network reconstructions. [file NPH-210-240-s001.pdf]

New Phytologist

***New Phytologist* Supporting Information**

Genetic architecture of wood properties based on association analysis and co-expression networks in white spruce

Mebarek Lamara, Elie Raherison, Patrick Lenz, Jean Beaulieu, Jean Bousquet and John MacKay

Article acceptance date: 13 October 2015

The following Supporting Information is available for this article:

Fig. S1 Plots of the 1694 white spruce trees on the plane of the two first eigenvectors derived from the principal component analysis (PCA).

Fig. S2 Overlap among sets of significantly associated genes (*P*<0.05) between the different traits as determined for latewood.

Fig. S3 Gene co-expression groups in white spruce (*Picea glauca*) according to Raherison *et al*. (2015) that were used for network reconstructions.

Table S1 Genes significantly associated with EW traits and their functions (see separate file)

Table S2 Genes significantly associated with LW traits and their functions (see separate file)

Table S3 The 93 selected genes that were connected to NAC-7, NAC-8, and to MYB1, MYB4, and MYB8 in the co-expression network and their functions (see separate file)

Methods S1 Candidate genes selection.

Methods S2 Information and formulas used for estimation of quantitative genetic parameters.

Methods S3 The hypergeometric test used for the evaluation of the over- and under-representation of candidate and significant genes in the co-expression groups.

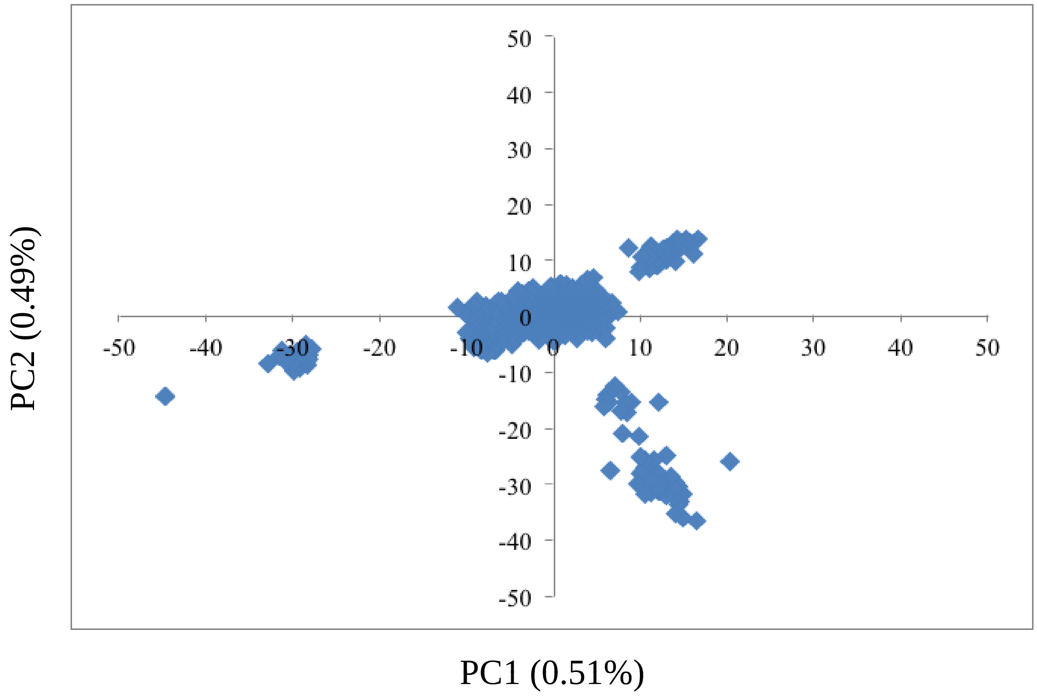

Fig. S1 Plots of the 1694 white spruce trees on the plane of the two first eigenvectors derived from the principal component analysis (PCA). Numbers within parentheses are the percentages of variation explained by the principal components, which are very low.

(a)

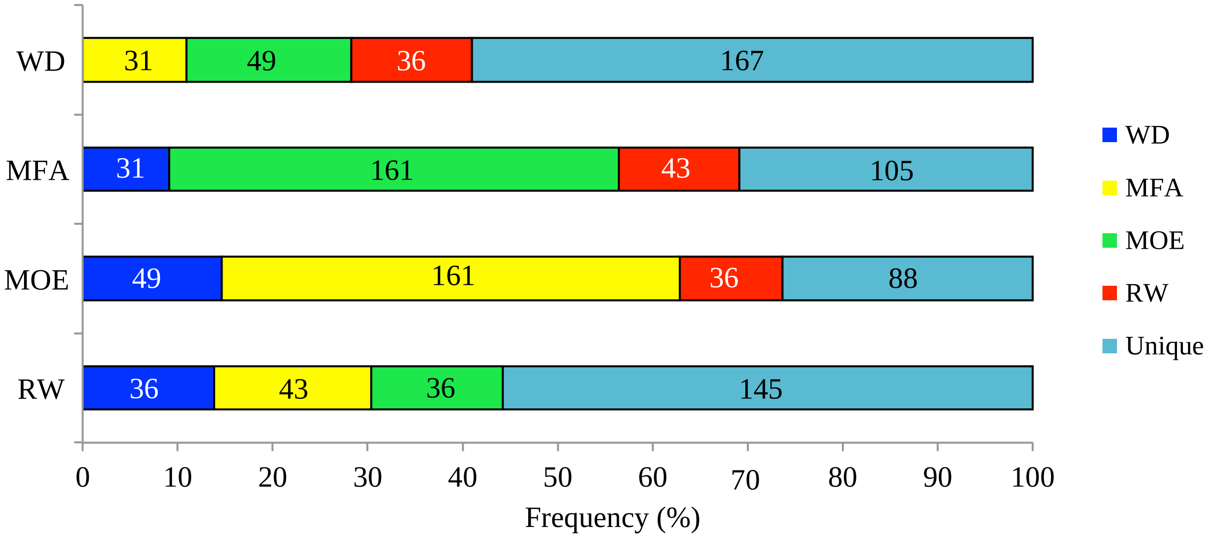

(b)

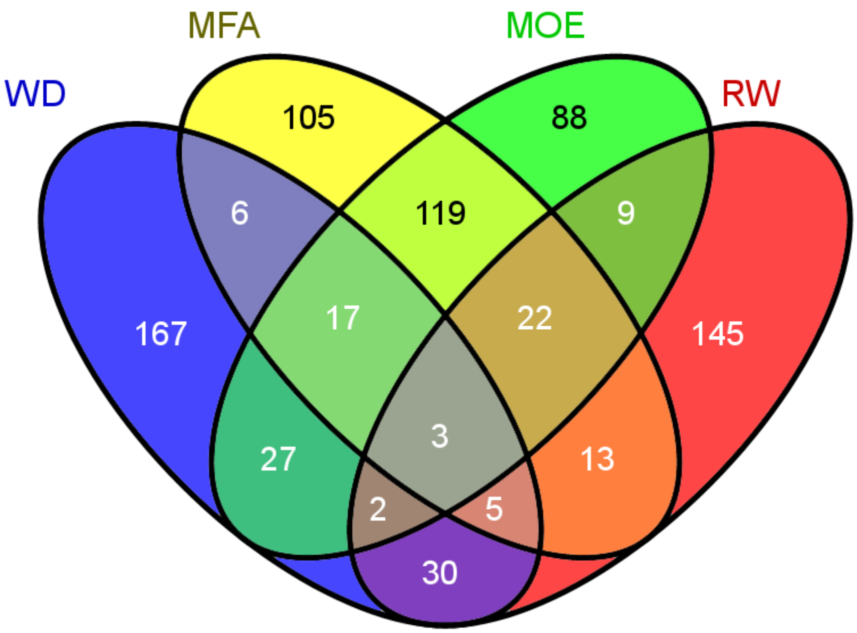

Fig. S2 Overlap among sets of significantly associated genes (*P*<0.05) between the different traits as determined for latewood. (a) Pairwise comparisons between traits, showing the numbers shared or unique genes; (b) Venn diagram showing the full extent of overlaps of associated genes between traits. (WD, wood density; MFA, microfibril angle; MOE, modulus of elasticity; RW, ring width).

(a)

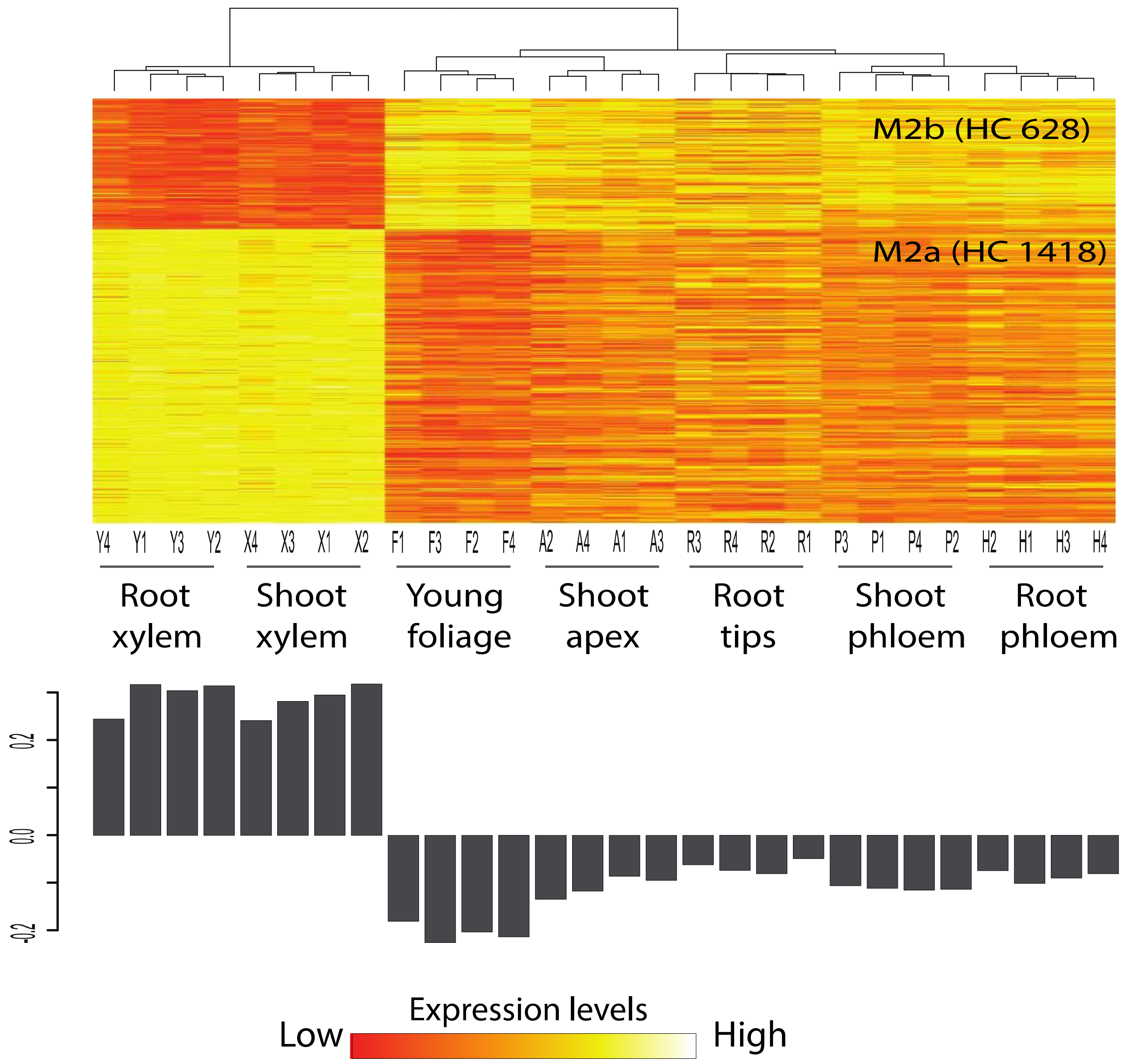
M2b (HC 628)
M2a (HC 1418)
Y4 Y1 Y3 Y2 X4 X3 X1 X2 F1 F3 F2 F4 A2 A4 A1 A3 R3 R4 R2 R1 P3 P1 P4 P2 H2 H1 H3 H4
Root xylem
Shoot xylem
Young foliage
Shoot apex
Root tips
Shoot phloem
Root phloem
0.2
0.0
-0.2
Expression levels
Low
High

(b)

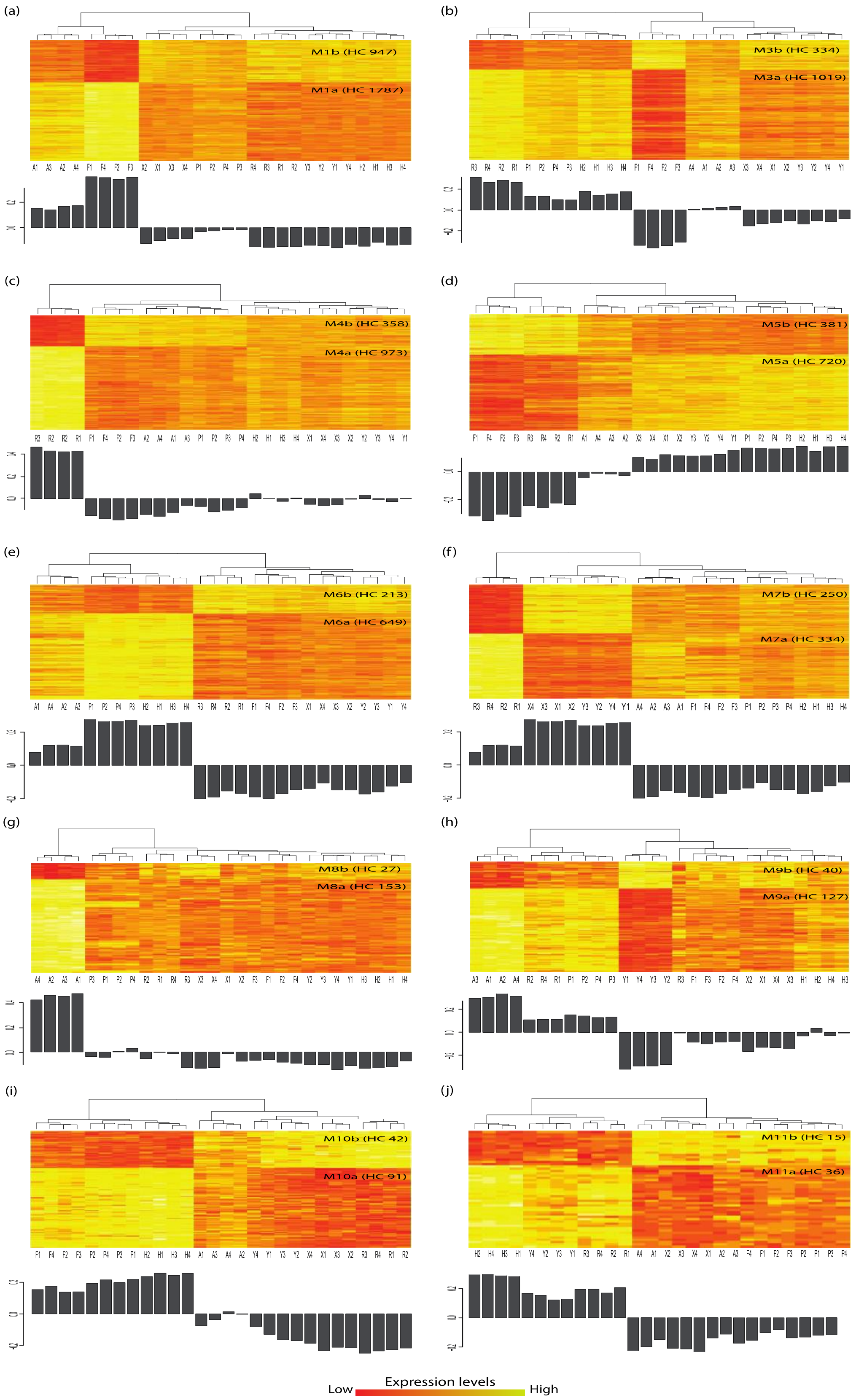

(a)
M1b (HC 947)
M1a (HC 1787)
(b)
M3b (HC 334)
M3a (HC 1019)
(c)
M4b (HC 358)
M4a (HC 973)
(d)
M5b (HC 381)
M5a (HC 720)
(e)
M6b (HC 213)
M6a (HC 649)
(f)
M7b (HC 250)
M7a (HC 334)
(g)
M8b (HC 27)
M8a (HC 153)
(h)
M9b (HC 40)
M9a (HC 127)
(i)
M10b (HC 42)
M10a (HC 91)
(j)
M11b (HC 15)
M11a (HC 36)
Low
Expression levels
High

Fig. S3 Gene co-expression groups in white spruce (*Picea glauca*) according to Raherison *et al.* (2015). (a) Heatmap of genes belonging to the M2 co-expression groups; (b) Heatmap of genes belonging to the other expression modules and co-expression groups M1 and M3–M11, expression modules; M1a,b and M3a–M11b, coexpression groups. (a–j) Heatmaps show transcript abundance (log2scale) of high-confidence (HC) variable genes in expression modules and co-expression groups (Raherison *et al.* 2015). Number in parentheses, number of high-confidence (HC) variable genes in the co-expression group. Rows (y-axis), genes which are grouped in M2 expression module; columns (x-axis), each of the four replicates of tissues (P1–4, shoot secondary phelloderm; Y1–4, root secondary xylem; R1–4, root tip; X1–4, shoot secondary xylem; F1–4, young foliage; A1–4, shoot apex; H1–4, shoot secondary phelloderm). Bar plots display the eigengene modules (or the first principal components) (Raherison *et al.* 2015).

References

Raherison E, Guiguère I, Caron S, Lamara M, MacKay J. 2015. Modular organization of the white spruce (*Picea glauca* (Moench) Voss) transcriptome reveals functional organization and evolutionary signatures. *New Phytologist* **207**: 172–187.

Methods S1 Candidate genes selection

The candidate genes used for association study (AS) in this report were selected from the white spruce gene database (Rigault *et al*., 2011) based on multiple criteria as described in Pavy *et al.* (2013). The candidate genes were selected to assembly SNP genotyping chip; therefore, for a given gene to be included, at least one SNP had to be known (for details, see Pavy *et al.*, 2013). A first set of criteria related to predicted functions based on sequence similarity. The gene functions that were target were very broad and deemed relevant for several traits including wood formation, growth, phenology, and adaptation to biotic and abiotic factors as indicated by database searching and scientific literature from Arabidopsis and poplar (e.g. Groover, 2005; Demura & Fukuda, 2007; Zhang *et al*., 2011). The selection was based on gene families and most members of a given family were considered. In total, 1868 gene families were represented (Pavy *et al.,* 2013). A second set of criteria, was gene expression evidence obtained in white spruce by integrating microarray profiling data sets and sequence analyses for phenology (El Kayal *et al*., 2011) and vascular tissue differentiation (Pavy *et al*., 2008b); however, gene expression data was not available for all of the genes and was not required for selection. Third, experimental evidence from investigations in white spruce were obtained from transgenic experiments with overexpression of R2R3-MYB genes, HD-zips and other transcription factors in spruce trees (Bomal *et al*., 2008; Bedon *et al*., 2010; Côté *et al*., 2010). Fourth, genes were selected based on their gene co-localization with QTLs for bud flush, bud set and height growth (Pelgas *et al*., 2011) and based on SNPs in gene coding sequences identified in outlier detection studies of local adaptation genes (Namroud *et al*., 2008), both in white spruce.

References

Bedon F, Bomal C, Caron S, Levasseur C, Boyle B, Mansfield SD, Schmidt A, Gershenzon J, Grima-Pettenati J, Séguin A *et al.* 2010. Subgroup 4 R2R3-MYBs in conifer trees: gene family expansion and contribution to the isoprenoid- and flavonoid-oriented responses. *Journal of Experimental Botany* **61**: 3847–3864.

Bomal C, Bedon F, Caron S, Mansfield SD, Levasseur C, Cooke JEK, Blais S, Tremblay L, Morency MJ, Pavy N *et al.* 2008. Involvement of *Pinus taeda* MYB1 and MYB8 in phenylpropanoid metabolism and secondary cell wall biogenesis: a comparative *in planta* analysis. *Journal of Experimental Botany* **59**: 3925–3939.

Côté CL, Boileau F, Roy V, Ouellet M, Levasseur C, Morency MJ, Cooke JE, Séguin A, MacKay J. 2010. Gene family structure, expression and functional analysis of HD-Zip III genes in angiosperm and gymnosperm forest trees. *BMC Plant Biology* **10**: 273.

Demura T, Fukuda H. 2007. Transcriptional regulation in wood formation. *Trends in Plant Science* **12**: 64–70.

El Kayal W, Allen CCG, Ju CJ-T, Adams E, King-Jones S, Zaharia LI, Abrams SR, Cooke JEK. 2011. Molecular events of apical bud formation in white spruce, *Picea glauca*. *Plant, Cell & Environment* **34**: 480–500.

Groover AT. 2005. What genes make a tree a tree? *Trends in Plant Science* **10**: 210–214.

Namroud M-C, Beaulieu J, Juge N, Laroche J, Bousquet J. 2008. Scanning the genome for gene single nucleotide polymorphisms involved in adaptive population differentiation in white spruce. *Molecular Ecology* **17**: 3599–3613.

Pavy N, Boyle B, Nelson C, Paule C, Giguère I, Caron S, Parsons LS, Dallaire N, Bedon F, Bérubé H *et al*. 2008b. Identification of conserved core xylem gene sets: conifer cDNA microarray development, transcript profiling and computational analyses. *New Phytologist* **180**: 766–786.

Pavy N, Gagnon F, Rigault P, Blais S, Deschênes A, Boyle B, Pelgas B, Deslauriers M, Clément S, Lavigne P *et al.* 2013. Development of high-density SNP genotyping arrays for white spruce (*Picea glauca*) and transferability to subtropical and nordic congeners. *Molecular Ecology Resources* **13**: 324–336.

Pelgas B, Bousquet J, Meirmans PG, Ritland K, Isabel N. 2011. QTL mapping in white spruce: gene maps and genomic regions underlying adaptive traits across pedigrees, years and environments. *BMC Genomics* **12**: 145.

Rigault P, Boyle B, Lepage P, Cooke J, Bousquet J, MacKay J. 2011. A white spruce gene catalogue for conifer genome analyses. *Plant Physiology* **157**: 14-28.

Zhang J, Elo A, Helariutta Y. 2011. *Arabidopsis* as a model for wood formation. *Current Opinion in Biotechnology* **22**: 293–299.

Methods S2 Information and formulas used for estimation of quantitative genetic parameters.

The following linear random model was used for quantitative genetic analyses:

$Y_{ijk} = \mu + \beta_j + F_i + B \text{ x } F_{ij} + e_{ijk}$,

where Y is the phenotypic observation on the kth tree, μ is the overall mean, β_j is the fixed effect of the jth block. F_i is the random effect of the ith half-sib family, B x F_{ij} is the random interaction between the ith family and the jth block, and e is the residual error term.

The narrow sense heritability (h_i^2) was calculated for each trait as:

$$h_i^2 = \frac{4\ x\ \sigma_f^2}{\sigma_f^2+\sigma_{bxf}^2+\sigma_e^2} \quad (1)$$

where σ_f^2 represents the estimated family variance, σ_{bxf}^2 the interaction between bloc and family effects, and σ_e^2 is the residual variance. The associated error of heritability ($S_{h_i^2}$) was estimated as:

$$S_{h_i^2} = \frac{4\ x\ s_{\sigma_f^2}}{\sigma_f^2+\sigma_{bxf}^2+\sigma_e^2} \quad (2)$$

where $s_{\sigma_f^2}$ is the standard error of the family variance.

Genetic and phenotypic correlations between wood traits were calculated with

$$r_{(x,y)} = \frac{COV_{x,y}}{\sqrt{\sigma_{(X)}^2 x \sigma_{(y)}^2}} \quad (3)$$

where $\sigma_{(X)}^2$ and $\sigma_{(y)}^2$ are the estimated additive genetic or phenotypic variance components for attributes x and y and $COV_{x,y}$ the co-variance between both traits. The co-variances were estimated in SAS using a multivariate approach, and associated errors for correlation estimates were calculated with the delta method as described by Lenz *et al.* (2013).

References

Lenz P, Auty D, Achim A, Beaulieu J, Mackay J. 2013. Genetic improvement of white spruce mechanical wood traits–early screening by means of acoustic velocity. *Forests* **4**: 575–594.

Methods S3 The hypergeometric test used for the evaluation of the over- and under-representation of candidate and significant genes in the co-expression groups

Hypergeometric test was used in this analysis to test for over or under-representation. For example, having a gene list, the distribution of these genes in the different co-expression groups, we want to know if the number of genes in each co-expression group is over- or under-represented.

Example: list of significantly associated genes with MFA trait in EW

Co-expression groups	F	K	N-K	n	N
M1a	18	249	2012	163	2261
M1b	22	249	2012	259	2261
M2a	57	249	2012	360	2261
M2b	9	249	2012	114	2261
M3a	16	249	2012	263	2261

Where:

F the number of significantly associated genes present in the co-expression group

K the total number of significantly associated genes with earlywood MFA

N the total number of candidate genes present in the 22 co-expression groups

n the number of candidate genes in the co-expression group

To test the genes in M2a co-expression group:

Test for over-representation

phyper(F-1, K, N-K, n, lower.tail= FALSE)
phyper(56, 249, 2012, 360, lower.tail= FALSE)
P-value = 0.001446824

Test for under-representation

phyper(F, K, N-K, n, lower.tail= TRUE);
phyper(57, 249, 2012, 360, lower.tail= TRUE)

P-value = 0.9991775

The results of *P*-values indicate that genes significantly associated with EW MFA are over represented in M2a (*P*-value <0.05).
